# Role of RelA-synthesized (p)ppGpp and ROS-induced mutagenesis in *de novo* acquisition of antibiotic resistance in *E. coli*

**DOI:** 10.1016/j.isci.2024.109579

**Published:** 2024-03-26

**Authors:** Wenxi Qi, Martijs J. Jonker, Wim de Leeuw, Stanley Brul, Benno H. ter Kuile

**Affiliations:** 1 Laboratory for Molecular Biology and Microbial Food Safety, Swammerdam Institute for Life Sciences, University of Amsterdam, Amsterdam, the Netherlands; 2 RNA Biology & Applied Bioinformatics, Swammerdam Institute for Life Sciences, University of Amsterdam, Amsterdam, the Netherlands

**Keywords:** Molecular biology, Microbiology

## Abstract

The stringent response of bacteria to starvation and stress also fulfills a role in addressing the threat of antibiotics. Within this stringent response, (p)ppGpp, synthesized by RelA or SpoT, functions as a global alarmone. However, the effect of this (p)ppGpp on resistance development is poorly understood. Here, we show that knockout of *relA* or *rpoS* curtails resistance development against bactericidal antibiotics. The emergence of mutated genes associated with starvation and (p)ppGpp, among others, indicates the activation of stringent responses. The growth rate is decreased in Δ*relA*-resistant strains due to the reduced ability to synthesize (p)ppGpp and the persistence of deacylated tRNA impeding protein synthesis. Sluggish cellular activity causes decreased production of reactive oxygen species (ROS), thereby reducing oxidative damage, leading to weakened DNA mismatch repair, potentially reducing the generation of mutations. These findings offer new targets for mitigating antibiotic resistance development, potentially achieved through inhibiting (p)ppGpp or ROS synthesis.

## Highlights

• Deletion of *relA* or *rpoS* results in a decelerated acquisition of resistance to bactericidal antibiotics
• Resistant strains exhibit mutations in genes linked to antibiotic resistance, oxidative stress, the SOS response, and the stringent response
• Knockout of *relA* leads to reduced ROS production and mitigated DNA oxidative damage
• Transcription levels of genes involved in DNA repair are diminished in Δ*relA*-resistant strains exposed to bactericidal antibiotics

## Graphical abstract

**Figure:**
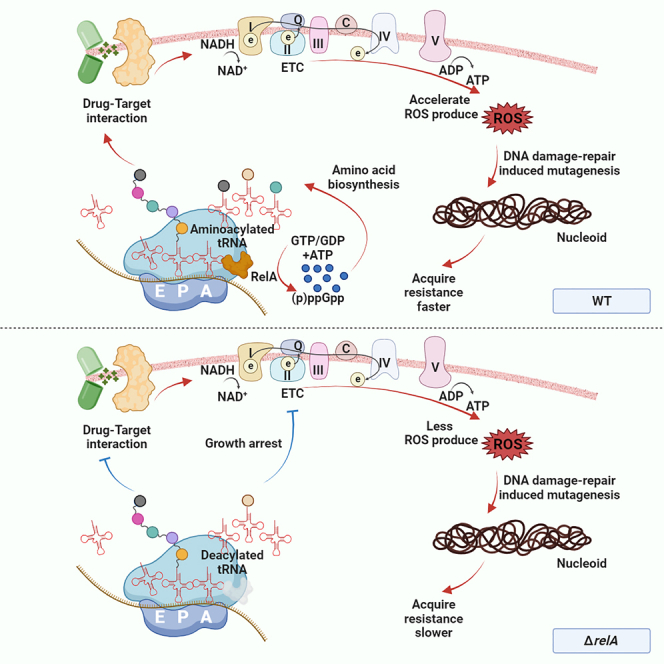

## Introduction

The spectrum of bacterial defense mechanisms against antimicrobials encompasses, but is not confined to, target alterations, upregulation of efflux pumps, reduction of cellular permeability, and modification of antibiotics.^1^ Such complicated mechanisms necessitate specific physiological activity within the cells. Stress caused by exposure to antibiotics and the subsequent increased protein synthesis triggers in bacteria the same stringent response as during nutrient starvation. This response is mediated by the synthesis of the signaling nucleotides guanosine tetraphosphate (ppGpp) and guanosine pentaphosphate (pppGpp), collectively termed (p)ppGpp.^2^^,^^3^ Bacterial resistance to antibiotics is caused by cellular adaptation in combination with genomic DNA mutations or acquisition of exogenous DNA.^4^ There is evidence indicating that molecular alterations seen as a result of the stringent response are related to the development of resistance mutations in both ways.^5^

The enzyme GDP/GTP pyrophosphokinase RelA plays a crucial role in the stringent response by catalyzing the synthesis of (p)ppGpp.^6^ Hence it is hypothesized that the knockout of *relA* results in the suppression of (p)ppGpp synthesis. Deletion of *relA* in *E. coli* results in reduced mutation rates in multiple amino acid auxotrophic strains, and a direct correlation has been established between the concentration of (p)ppGpp and the mutation rate.^7^^,^^8^ Furthermore, the involvement of (p)ppGpp extends to the regulatory control of class 1 integron integrases which enable bacteria to express and capture antibiotic resistance gene cassettes under starvation-induced stringent response in biofilms.^9^

The regulatory impact of (p)ppGpp, accomplished through its direct interaction with RNA polymerase, manifests itself in the modulation of transcription initiation at specific gene promoters. Additionally, (p)ppGpp is involved in modulating the production and activity of the RNA polymerase sigma factor RpoS, which serves as the master transcriptional regulator of the general stress response.^10^ HipA and HipB are components of a type II toxin-antitoxin (TA) system. It has been demonstrated that HipA expression activates ppGpp synthesis mediated by RelA.^11^ On the other hand, HipB functions as an antitoxin, counteracting the toxic effects of the cognate toxin HipA.^12^ Consequently, the knockout of *hipB* may potentially result in an elevated level of ppGpp. This study addresses the role of stringent response mediated stress responses on antibiotic resistance development upon long-term exposure to sub-lethal levels of antibiotics, by means of evolution experiments on four (p)ppGpp associated *E. coli* knockout strains Δ*relA*, Δ*rpoS*, Δ*hipA*, and Δ*hipB*.

Reactive oxygen species (ROS) produced upon exposure to sublethal levels of bactericidal antibiotics affect development of resistance according to a hormesis mechanism.^13^^,^^14^ That is, high levels of ROS kill cells, while sub-lethal levels of ROS affect cellular DNA inducing mutations that may be beneficial in promoting the formation of antibiotic resistance.^15^ Besides their specific antibiotic-target interactions, bactericidal antibiotics, such as β-lactams, quinolones, and aminoglycosides, stimulate oxidation of NADH via the electron transport chain.^16^ As a consequence, superoxide formation is also enhanced, which in turn promotes hydroxyl radical formation via the Fenton reaction.^17^^,^^18^ These byproducts, collectively called ROS, are very reactive with cellular components, especially DNA, for example by oxidizing guanine to 8-hydroxy-2′-deoxyguanosine (8-HOdG), which may increase mutation rates.^19^ The oxidative response induced by ROS and the stringent response triggered by (p)ppGpp are both metabolic feedback loops by which cells respond to various stresses. The connection between these two stress responses and their implications for acquisition of resistance under antibiotic exposure remain to be elucidated.

In this investigation, we quantified the ROS levels during antibiotic resistance development within (p)ppGpp-synthesis mutant strains, simultaneously assessing the inflicted damage due to ROS and the subsequent repair processes. We propose a mechanism for the interplay between the stringent stress response and oxidative stress responses and corroborate the association between ROS-mediated hormesis and the evolution of antimicrobial resistance, particularly at relatively moderate ROS levels.

## Results

### Lower rates of resistance development in *relA* or *rpoS* knockout strains

**Figure 1:**
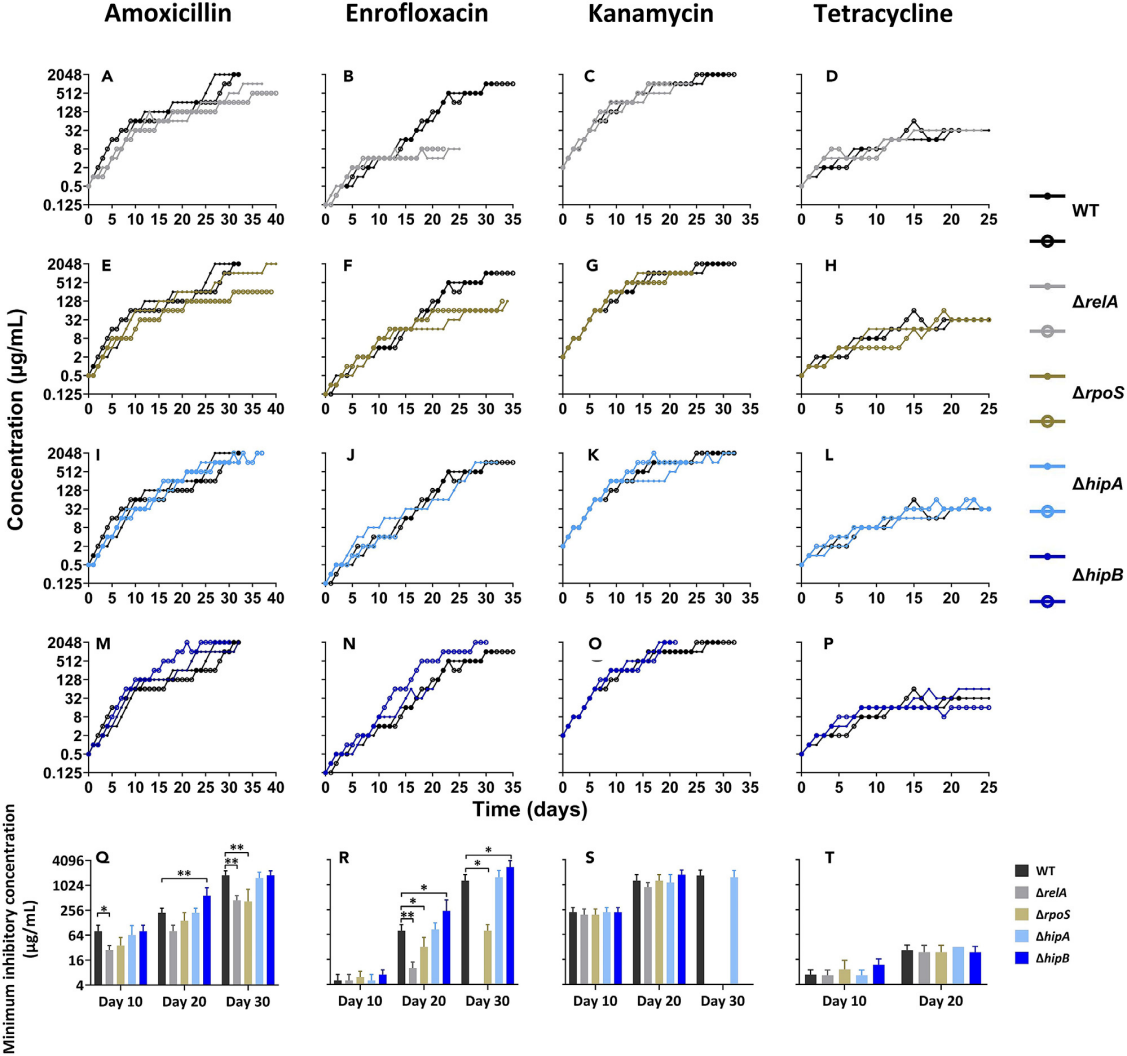
Effect of *relA*, *rpoS*, *hipA*, and *hipB* knockouts on antibiotics resistance development (A–P) Antibiotic resistance development was evaluated in *E. coli* wild-type MG1655 (WT) and single gene knockout strains Δ*relA*, Δ*rpoS*, Δ*hipA*, and Δ*hipB* against amoxicillin (A, E, I, and M), enrofloxacin (B, F, J, and N), kanamycin (C, G, K, and O), and tetracycline (D, H, L, and P). The x axis represents the duration of evolution in days, while the y axis represents the concentration of acquired resistance. (Q–T) Comparison of minimum inhibitory concentration (MIC) at day 10, day 20, and day 30 for each strain (WT, Δ*relA*, Δ*rpoS*, Δ*hipA*, and Δ*hipB*) against amoxicillin (Q), enrofloxacin (R), kanamycin (S), and tetracycline (T) during the process of antibiotic resistance acquisition. Data are presented as means ± SD, statistical significance was determined using a one-way ANOVA, n ≥ 3, ∗*p* < 0.05, ∗∗*p* < 0.01.

To investigate the effect of (p)ppGpp on the acquisition of *de novo* antibiotic resistance, we exposed fully susceptible *E. coli* wild-type and four single-gene knockout strains Δ*relA*, Δ*rpoS*, Δ*hipA*, and Δ*hipB* to stepwise-increasing sub-lethal concentrations of four antibiotics (Figure 1). The resistance evolution experiments started at one-quarter of the minimum inhibitory concentration (MIC) for amoxicillin (0.5 μg/mL), enrofloxacin (0.125 μg/mL), kanamycin (2 μg/mL), and tetracycline (0.5 μg/mL).

During the early stages of exposure to amoxicillin, the resistance acquisition rate of the Δ*relA* was lower than that of the WT strains (Figure 1A). At day 10 the MIC of the Δ*relA* strain made resistant to amoxicillin was significantly lower than that of the WT (Figure 1Q). The final resistance concentrations of the Δ*relA* were in two independent experiments 512 μg/mL and 1,024 μg/mL, respectively, versus 2,048 μg/mL for the wild-type strains (Figure 1A). Similar to the Δ*relA*, the MIC of the Δ*rpoS* at day 30 was significantly lower than that of the wild-type strains (Figure 1Q). The maximum resistance concentration of one replicate of the Δ*rpoS* was 256 μg/mL, and both replicates reached their final resistance concentrations later than the WT-resistant strain (Figure 1E). There were no apparent differences observed in the development of amoxicillin resistance between the wild-type and Δ*hipA* strains (Figure 1I). Resistance development of Δ*hipB* progressed at a higher rate than that of the wild-type strains during the middle stages, but the final level equaled that of the wild-type (Figure 1M). The MIC of the Δ*hipB* at day 20 was higher than that of the WT strain (Figure 1Q).

During enrofloxacin exposure, clear differences between the strains could be observed after day 10 (Figures 1B–1F, 1J, and 1N). At day 20, the MIC of the Δ*relA*-resistant strain and Δ*rpoS*-resistant strain were significantly lower than the WT-resistant strain, and all these strains exhibited lower MIC values compared to the Δ*hipB*-resistant strain (Figure 1R). The final resistance concentrations of Δ*relA* and Δ*rpoS* were 8 μg/mL and 128 μg/mL, respectively, considerably lower than the wild-type strains’ concentration of 1,024 μg/mL (Figures 1B and 1F). One replicate of the Δ*hipA* ceased to grow at day 13, but the other one exhibited a similar resistance acquisition rate and MIC as the wild-type strains (Figures 1J and 1R). Similarly, resistance of one replicate of the Δ*hipB* stopped increasing at day 20 of 64 μg/mL. However, the other replicate showed a higher resistance acquisition rate than the wild-type strains, and its final resistance concentrations were double those of the WT-resistant strains (Figures 1N and 1R).

During kanamycin exposure, the rate of resistance acquisition and the MIC at days 10 and 20 were relatively consistent across all strains (Figures 1C–1G, 1K, 1O, and 1S). However, the maximum resistance concentrations of Δ*relA* and Δ*rpoS* were both 1,024 μg/mL, which was half that observed in the WT-resistant strains (Figures 1C and 1G). The *hipA* knockout had no meaningful effect on the resistance development (Figure 1K). Remarkably, the Δ*hipB* reached kanamycin resistance concentrations of 2,048 μg/mL at day 20, which was faster than the wild-type strains (Figure 1O).

In the case of the bacteriostatic antibiotic tetracycline, the strains reached their final resistance concentrations at 32 μg/mL, which was low compared to the bactericidal antibiotics (Figures 1D–1H, 1L, and 1P). Additionally, the resistance acquisition rate, and MIC at day 10 or day 20 were all roughly the same in each strain (Figure 1T).

### Mutations in stress response genes accompany antibiotic resistance

At the end of the antibiotic resistance evolution experiments, the genomic DNA of the final resistant strains was sequenced entirely to identify mutations that may influence development of resistance. The mutations that accompanied antibiotic resistance were clustered into different groups according to their functions as defined by the Comprehensive Antibiotic Resistance Database and UniProt (Tables 1, 2, 3, 4, and 5).^20^^,^^21^ The mutations that relate to metabolism or that are functionally unknown are given in the appendix (Table S1).

**Table 1:** Mutations associated with resistance development after amoxicillin exposure

Functions	Genes	WT	Δ*relA*_1	Δ*relA*_2	Δ*rpoS_*1	Δ*rpoS_*2	Δ*hipA*_1	Δ*hipA*_2	Δ*hipB*_1	Δ*hipB*_2
Antibiotic inactivation	*ampC* (promoter)	a34_32del a73_72insA	a38C>A a70C>T	a38C>A a70C>T	a30G>T a77G>T a81dupT	a37G>C a40C>G	a25C>A a83dupA	a38C>G a75dupC	a38delC a70C>T	a70C>T a91T>A
Reduced permeability	*envZ*			A175V	V241G	I86S	R397C	I19del T15P		A175E
	*ompC*	Q171stop		D39fs	Y74stop	D33fs	10_91del	D134dup		Q258stop
Efflux pumps	*acrB*					S608L	S135R		F628L	
	*soxR*						L149fs			
	*cpxA*	Q202stop		16_17insLA		19_20 insVLML	16_17del	D162N		
Target alteration	*ftsI*			V545I	A513S	P311A V545I				
	*mrdA*					A586V				
	*rpoA*			S299Y	V257L			L234dup		
	*rpoB*						469_470 delinsG			
	*rpoC*	N341T								
	*rpoD*		D445A	D445A			D445V		A444V	
OMP	*ompX*				a98G>A	a99delT				
Antitoxin	*chpS*		del							
Toxin	*ghoT*							L34S A37T A37G		
DNA protection	*dps*			a134C>A						
Elongation	*tufA*								A183fs	
Chaperonin	*groL*								540_541 insMGG	
Regulator	*slyA*									G6fs
SIM	*hfq*									N48K

OMP, outer membrane protein; SIM, stress-induced mutagenesis.

a Upstream mutation.

**Table 2:** Copy numbers of gene amplification regions including *ampC* after amoxicillin exposure

Strains	Length (kb)	Copy numbers (times)	Upstream gene	Downstream gene
WT	2.6	17	*frdD*	*ecnB*
Δ*relA*_1	11.5	18	*mscM*	*epmB*
Δ*relA*_2	11.5	10	*mscM*	*epmB*
Δ*rpoS_*1	83	16	*hflX*	*phnI*
Δ*hipA*_1	67	15	*frdA*	*rpiB*
Δ*hipA*_2	10.5	116	*yjeM*	*yjeJ*
Δ*hipB*_1	9	100	*yjeN*	*ecnA*
Δ*hipB*_2	8.5	150	*frdA*	*yjeI*

In the table, the size of the fragment, the amplification factor, and upstream and downstream of the genes contained in the fragment are listed for each strain tested.

**Table 3:** Mutations associated with resistance development after enrofloxacin exposure

Functions	Genes	WT	Δ*relA*_1	Δ*relA*_2	Δ*rpoS_*1	Δ*rpoS_*2	Δ*hipA*_1	Δ*hipA*_2	Δ*hipB*_1	Δ*hipB*_2
Target alteration	*gyrA*	D87N S83L	G81C	S83L	D87Y	S83L	D87G S83A	D82G	D87Y	D87G S83L
	*gyrB*						S464F			S464F
	*parC*	S80R					S80I D79V			K278M K278stop K277N S80I
	*parE*	I437F								E460K
	*rpoA*	A230dup								
	*rpsA*					N236dup				
Efflux pumps	*acrR*		a30T>C	Q78K	F119fs	R105fs	43_45del	Q78K	E103fs	E104fs
	*acrA*	a95delT								
	*phoQ*	E233del								
	*soxR*				a25delAA	D137G	S128del		R127del	
DNA repair	*dinG*	A29fs								
	*yoaA*	M554fs								
(p)ppGpp synthase	*spoT*			114_121 dup						
DNA protection	*dps*				M65I					
SOS stress	*sulA*						a22A>G			
Elongation	*lepA*								N417Y	
Oxidative stress	*oxyR*									A213fs A288fs

a Upstream mutation.

**Table 4:** Mutations associated with resistance development after kanamycin exposure

Functions	Genes	WT	Δ*relA*_1	Δ*relA*_2	Δ*rpoS_*1	Δ*rpoS_*2	Δ*hipA*_1	Δ*hipA*_2	Δ*hipB*_1	Δ*hipB*_2
Reduced permeability	*sbmA*	F6fs	W53stop		L21stop	G256stop	18_19 insT	314_316 del	V207fs	
	*trkH*			L80Q						
Efflux pumps	*kdpD*	660_662 del							D460V	
	*cpxA*					D245Y				
Target alteration	*pgsA*			V75E						P6L
	*fusA*	T393I	T393I	T393I	R146C	R59C	P610L	T393I	P121R	T393I
	*rplF*				Y157C					
	*rplL*				44_49dup V41E					
ATP synthase	*atpG*	K248fs					Q240dup		M244fs	240_245 del
	*atpB*								A267fs	
Oligopeptide transport	*oppB*				W166fs				S196P E199G	L2fs
	*oppD*	V203fs		M70fs		A221fs				
	*oppF*						Q202K	V204del		
(p)ppGpp synthase	*spoT*			G315S

**Table 5:** Mutations associated with resistance development after tetracycline exposure

Functions	Genes	WT	Δ*relA*_1	Δ*relA*_2	Δ*rpoS_*1	Δ*rpoS_*2	Δ*hipA*_1	Δ*hipA*_2	Δ*hipB*_1	Δ*hipB*_2
Reduced permeability	*envZ*	167_168 insMLLAI							L88F	
	*ompF*			Q225stop						L240fs
Target protection	*rpsJ*	V57L		V57L					V57L	V57L
Efflux pumps	*acrB*						Q569L	M573I		
	*acrR*				S31Y		a41delA	S31Y		
	*rob*								246_247 delinsS	
	*marR*					V84E				
	*mprA*					53_54insT				
Target alteration	*rpoB*							30_31insPI		
	*rpoC*						215_220del			
	*rpoD*					T459A				
OMP	*mlaA*		44_45del	44_45del			44_45del	44_45del		
(p)ppGpp synthase	*spoT*		G315S	G315S						
Anti-sigma factor	*rseB*							D171fs		

OMP, outer membrane protein.

a Upstream mutation.

Amoxicillin-resistant strains displayed shared mutations within the *ampC* promoter region (Table 1). These mutations likely contribute to an upregulation in the levels of the β-lactamase AmpC.^22^^,^^23^ Additionally, all amoxicillin-resistant strains except the Δ*rpoS_*2 contained *ampC* amplification regions, varying in size and copy numbers, consistent with our previous findings (Table 2).^24^^,^^25^^,^^26^ Mutations in *envZ* and *ompC*, which encode the sensor histidine kinase EnvZ (OmpB) and outer membrane porin OmpC, respectively, were frequently observed.^27^ These mutations may result in reduced antibiotic entry into the cells.^28^ Occasional mutations were also observed in genes associated with efflux pumps or target alterations, albeit less frequently. Notably, a *chpS* deletion mutation was identified in one replicate of the Δ*relA* strain, and an upstream mutation in the *dps* gene occurred in another replicate. The antitoxin coding gene *chpS* together with the toxin gene *chpB* belong to a type II toxin-antitoxin (TA) system, potentially involved in the regulation of cell growth.^29^ *dps* codes for a DNA protection protein for starvation response, it protects DNA against multiple stresses, including oxidative stress.^30^ Among the Δ*hipA*_2-resistant strain, three missense variations were found in the toxin coding gene *ghoT*. Together with the antitoxin gene *ghoS*, this type V TA system plays a role in limiting cell growth during antibacterial stress.^31^ In the Δ*hipB*_2-resistant strain, a mutation was observed in the *hfq* gene, which encodes an RNA-binding protein. Hfq is part of a gene network that facilitates stress-induced mutagenesis (SIM) in *E. coli*.^32^

Enrofloxacin targets DNA gyrase and topoisomerase IV thereby inhibiting bacterial DNA synthesis.^33^ The shared mutations occurring in the DNA gyrase subunit A coding gene, *gyrA* (Table 3), and located on the quinolone resistance-determining region (QRDR), result in reduced affinity for quinolones.^34^ Resistant strains of the WT, Δ*hipA*_1, and Δ*hipB*_2 acquired mutations in *parC*, *gyrB*, or *parE*, enhancing their resistance and enabling them to reach higher concentrations of enrofloxacin resistance compared to other strains (Figures 1J and 1N). Another resistance mechanism these strains evolved operated through efflux pumps that expel the antibiotic. The main mutated gene associated with this mechanism was *acrR*, which encodes the HTH-type transcriptional regulator AcrR, known as a repressor of the AcrAB-TolC multidrug efflux complex.^35^ In the WT-resistant strain, DNA repair-related DNA helicase coding genes *dinG* and *yoaA* were mutated.^36^^,^^37^ Interestingly, the (p)ppGpp synthase/hydrolase coding gene *spoT* exhibited a mutation in the Δ*relA*_2, possibly activating the stringent response in these cells.^38^ The *dps* mutation found in the amoxicillin-resistant ΔrelA_2 appeared again but in a different position in the enrofloxacin-resistant strain Δ*rpoS*_1. In the Δ*hipA*_1 strain, a mutation occurred in the cell division inhibitor SulA, a component of the SOS system.^39^ Furthermore, two mutations were identified in the hydrogen peroxide-inducible gene activator coding gene *oxyR* in the Δ*hipB*_2 resistant strain, suggesting that the strain is under oxidative stress caused by ROS.^40^

The gene mutated in each of the kanamycin-resistant strains was *fusA*, with the predominant mutation being T393I (Table 4). The *fusA* gene encodes the elongation factor G, which plays a crucial role in ribosomal translocation during translation elongation.^41^ These mutations in *fusA* may contribute to the reduced binding efficiency of kanamycin. Another frequently mutated gene, *sbmA*, encodes an inner-membrane transport protein that in mutated form has been proven to impede the uptake of antimicrobial peptides.^42^^,^^43^ While the function remains unknown, mutations in the oligopeptide transport protein genes *oppB*, *oppD*, or *oppF* have been implicated in kanamycin resistance.^44^^,^^45^ Interestingly, a mutation in the bifunctional (p)ppGpp synthase/hydrolase *spoT* gene was also identified in the kanamycin-resistant strain Δ*relA*_2.

There was no shared mutated gene observed among all tetracycline-resistant strains (Table 5). However, a V57L mutation in the ribosomal subunit coding gene *rpsJ* was observed in the WT, Δ*relA*_2, Δ*hipB*_1, and Δ*hipB*_2 strains. This mutation may protect the antibiotic target. Furthermore, a 44_45del mutation within the outer membrane lipoprotein coding gene *mlaA* was observed in the Δ*relA* and Δ*hipA* resistant strains. A G315S mutation in the *spoT* gene that codes for a key enzyme of the stringent response was observed both in the kanamycin-resistant strain Δ*relA*_2 and in the tetracycline-resistant Δ*relA* strains (Table 4). The tetracycline-resistant strains shared several common mutated genes compared to other antibiotic-resistant strains. Mutations in the *envZ* gene and the RNA polymerase subunit coding genes *rpoBCD* were observed under both amoxicillin and tetracycline treatment. These mutations may cause reduced antibiotic permeability, and alterations in antibiotic targets, respectively (Table 1). Mutations were also detected in the efflux pump regulator gene *acrR* and the multidrug efflux pump subunit coding gene *acrB* under tetracycline exposure, while an *acrA* mutation was identified during enrofloxacin treatment (Table 3). These findings suggest that these mutations may not be specific to a particular antibiotic.

### Reduced oxidative stress and DNA damage in Δ*relA*-resistant strains during exposure to bactericidal antibiotics

**Figure 2:**
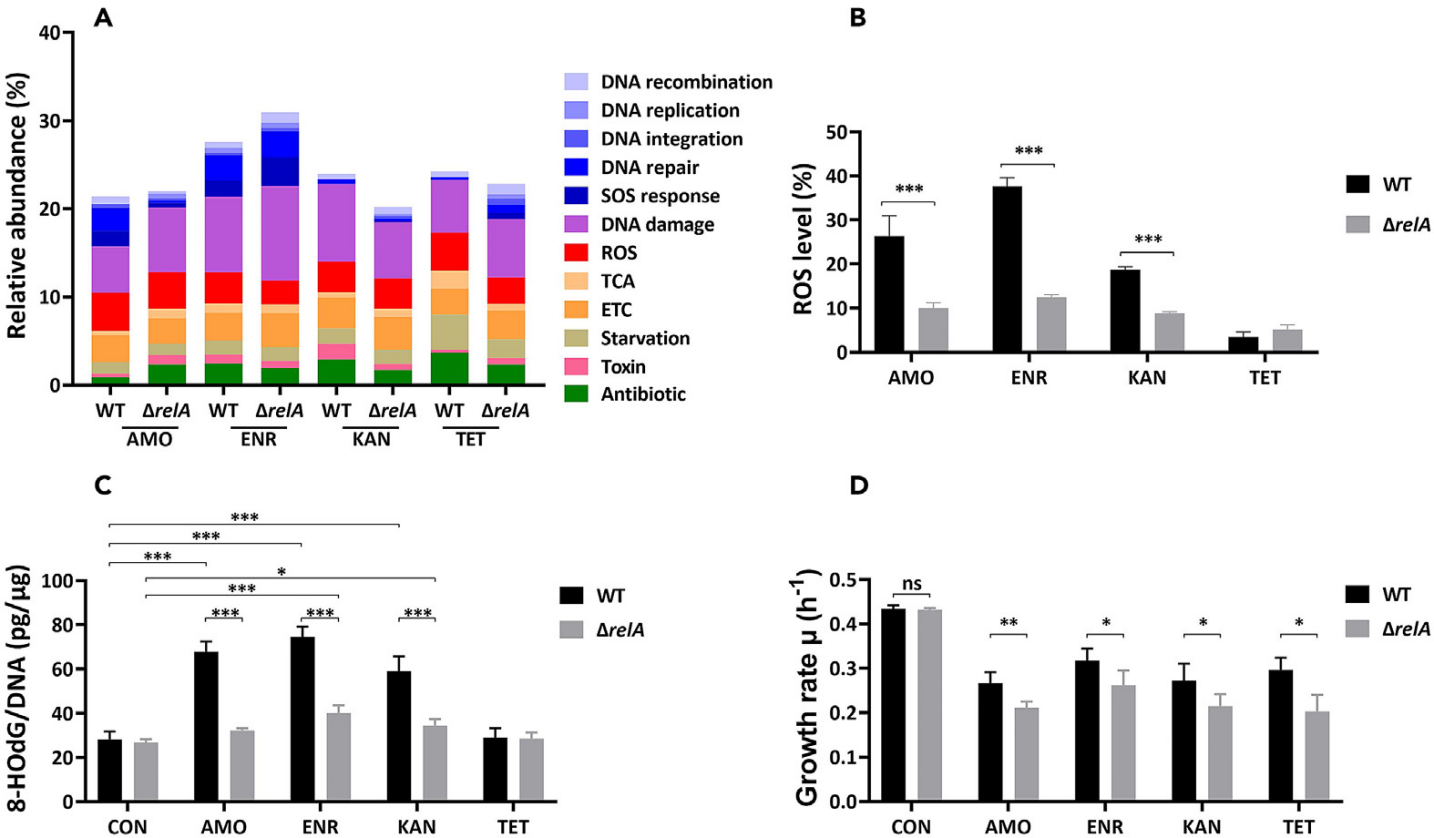
Differential oxidative stress and DNA damage in Δ*relA* resistant strains during bactericidal antibiotics exposure (A) Transcriptomic analysis of *E. coli* resistant WT and Δ*relA* strains after antibiotics exposure, revealing the relative abundance of regulated genes. Functional cluster according to the Gene Ontology Biological Process (GO-BP) and based on log_2_ fold change values higher than 2 and lower than −2. The selected clusters refer to whole genome sequencing results and are depicted in the scale bar. The abundance shows the percentage of genes within each selected cluster relative to the total number of upregulated and downregulated genes with a log_2_ fold change cutoff higher than 2. ROS, reactive oxygen species; TCA, citric acid cycle; ETC, electron transport chain; AMO, amoxicillin-treated; ENR, enrofloxacin-treated; KAN, kanamycin-treated; TET, tetracycline-treated. (B) Assessment of ROS production levels in resistant *E. coli* WT and Δ*relA* strains following exposure to maximum concentrations of each antibiotic. The Y axis represents the percentage of ROS-producing cells within each population. Data are presented as means ± SD. Statistical significance was determined using a one-way ANOVA, *N* = 3, ∗*p* < 0.05, ∗∗*p* < 0.01, ∗∗∗*p* < 0.001. (C) Evaluation of 8-HOdG production levels in resistant *E. coli* WT and Δ*relA* strains after treatment with maximum antibiotic concentrations. The Y axis represents the 8-HOdG concentrations divided to DNA concentrations. Data are presented as means ± SD. Statistical significance was determined using a one-way ANOVA, *N* = 3, ∗*p* < 0.05, ∗∗*p* < 0.01, ∗∗∗*p* < 0.001. CON, untreated naive cells. (D) Growth rate of resistant *E. coli* WT and Δ*relA* strains after treatment with maximum antibiotic concentrations. Data are presented as means ± SD. Statistical significance was determined using a one-way ANOVA, *N* = 3, ∗*p* < 0.05, ∗∗*p* < 0.01, ns, not significant. CON, naive cells without antibiotics.

In these *de novo* antibiotic resistant strains, mutations were observed not only in genes directly associated with drug resistance but also in a variety of genes involved in regulating cellular stress responses (Tables 1, 2, 3, 4, and 5). These stress responses, including oxidative stress response, stringent response, and SOS stress response, have been documented in relation to the development of antibiotic resistance.^5^^,^^15^^,^^46^^,^^47^^,^^48^ To further investigate that notion, we performed RNA differential quantification on the Δ*relA*-resistant strains that exhibited a slower evolution rate compared to the WT-resistant strains during bactericidal antibiotic treatment. (Figures 1A–1D). The differentially expressed genes associated with these stress responses were categorized according to the Gene Ontology Biological Process (GO-BP), with a log_2_ fold change cutoff of 2 applied to select the relevant genes (Figure 2A). Notably, a substantial proportion of the differentially expressed genes were related to the response to oxidative stress and DNA damage (Figure 2A). Therefore, we measured the ROS production levels and oxidation-mediated DNA damage characteristic 8-HOdG level in these strains (Figures 2B and 2C).

Among all the Δ*relA* and WT final resistant strains, the ROS production levels in cells exposed to bactericidal antibiotics were higher than in cells exposed to the bacteriostatic antibiotic tetracycline (Figure 2B). In addition, significantly lower ROS production levels were detected in the Δ*relA*-resistant strains compared to WT-resistant strains under exposure to bactericidal antibiotics amoxicillin, enrofloxacin, and kanamycin (Figure 2B). Moreover, the 8-HOdG production level was significantly increased in the WT-resistant strains exposed to the maximum concentrations of bactericidal antibiotics compared to the untreated naive cells (Figure 2C). In the Δ*relA*-resistant strains, enrofloxacin or kanamycin treatment caused clearly higher 8-HOdG production levels. Similar to the ROS production level, the 8-HOdG production level in Δ*relA*-resistant strains was significantly lower than in WT-resistant strains under bactericidal antibiotics treatment.

We assumed that ROS levels decreased as a result of the growth rate reduction caused by the knockout of the (p)ppGpp synthesis gene, *relA*. This lower growth rate would lead to a decline in respiratory chain activity, subsequently resulting in reduced ROS production. To verify this hypothesis, we measured the growth rates of the WT and Δ*relA* final resistant strains under maximum antibiotic concentrations (Figure 2D). There was no difference in growth rates between the WT and Δ*relA* naive strains without antibiotic in the medium. However, the growth rate of the Δ*relA*-resistant strains was significantly decreased compared to that of the WT-resistant strains when exposed to each antibiotic. In summary, our study findings indicate that the Δ*relA*-resistant strains exhibit reduced DNA damage caused by ROS during exposure to bactericidal antibiotics, primarily due to the decreased growth rate induced by the *relA* knockout.

### Knockout of *relA* resulted in decreased transcription levels of DNA repair genes during exposure to bactericidal antibiotics

**Figure 3:**
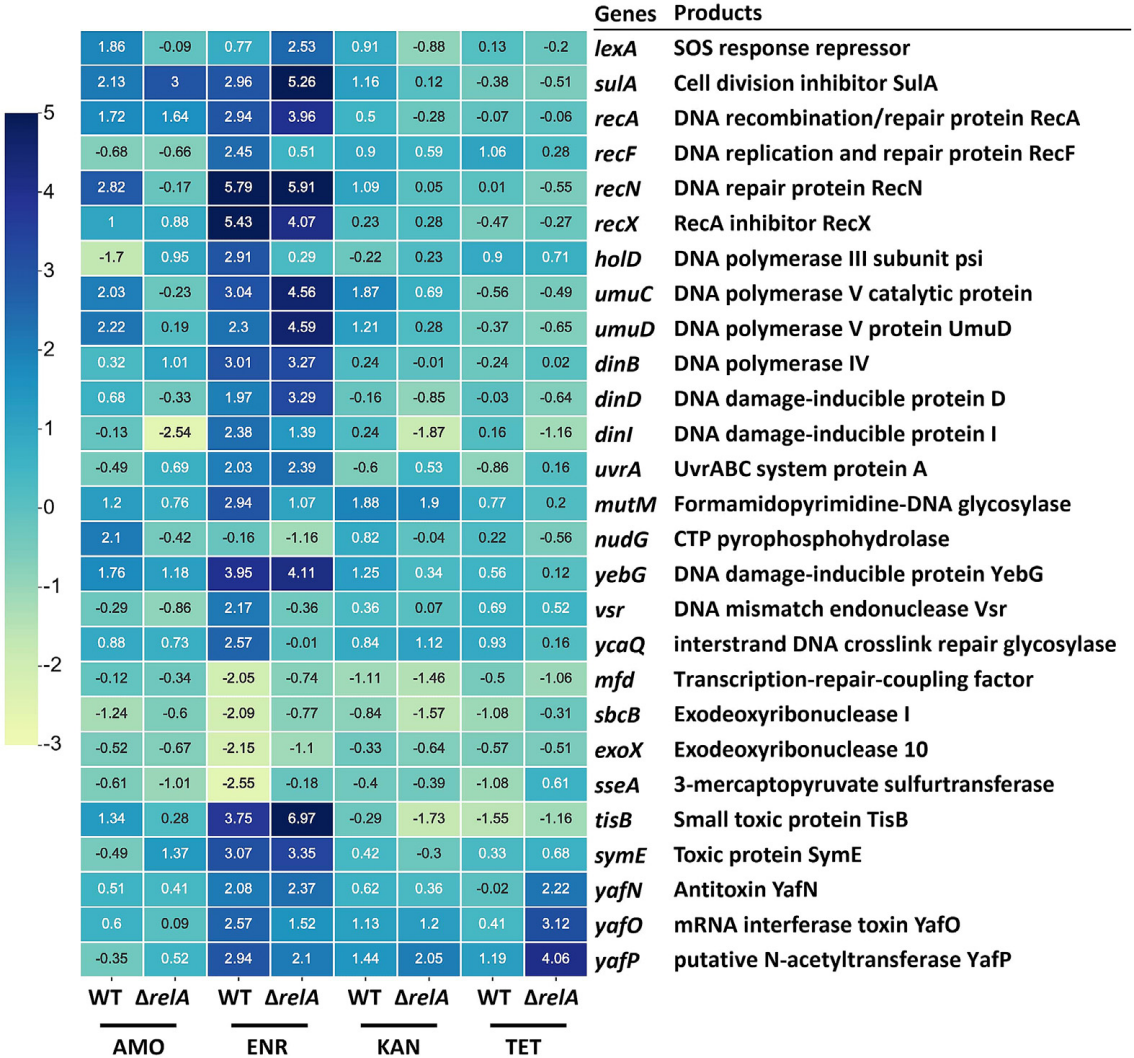
Gene transcription levels of DNA damage-repair-associated genes The heatmap represents the log_2_ fold change values of gene expression, with blue color indicating upregulated genes and yellow color indicating downregulated genes. Genes associated with DNA damage-repair were selected based on a log_2_ fold change cutoff greater than 2 in any treatment group. The right column provides information on the regulated genes and their corresponding functions.

In response to DNA damage, cells activate various mechanisms to detect and repair that damage. To identify the differentially expressed genes involved in DNA damage repair, we focused on the WT and Δ*relA*-resistant strains under maximum concentrations of antibiotics (Figure 2A). The Δ*relA* mutant was chosen for comparison with the WT as it lacks a key enzyme of the stringent response. Genes associated with DNA repair with a log_2_ fold change greater than 2 compared to untreated naive cells were selected and are represented in a heatmap (Figure 3).

During amoxicillin treatment, both the WT and Δ*relA*-resistant strains exhibited upregulation of *sulA* and *recA*, with similar expression levels. Transcription levels of *lexA*, *recN*, *umuC*, *umuD*, *mutM*, *nudG*, *yebG*, and *tisB* were upregulated in the WT but attenuated in the Δ*relA*-resistant strain. Enrofloxacin treatment induced the highest number of differentially expressed genes, including *sulA*, *recANX*, *umuCD*, *dinBDI*, *uvrA*, *yebG*, *tisB*, *symE*, and *yafNOP*, and caused the highest upregulation levels in both the WT and Δ*relA*-resistant strains. The transcription levels of *recF*, *holD*, *mutM*, *vsr*, and *ycaQ* were lower in the Δ*relA*-resistant strain, while *lexA* exhibited higher expression compared to the WT during exposure to enrofloxacin. Kanamycin treatment resulted in minor differences between the WT and Δ*relA*-resistant strains. Even so, the transcription level of *lexA*, *sulA*, *recN*, *umuCD*, and *yebG* were higher in the WT. Under tetracycline treatment, *recF* exhibited higher upregulation level in the WT compared to Δ*relA*, whereas in *yafNOP* displayed the opposite trend. Other genes showed relatively similar expression levels between the two tetracycline-resistant strains.

## Discussion

**Figure 4:**
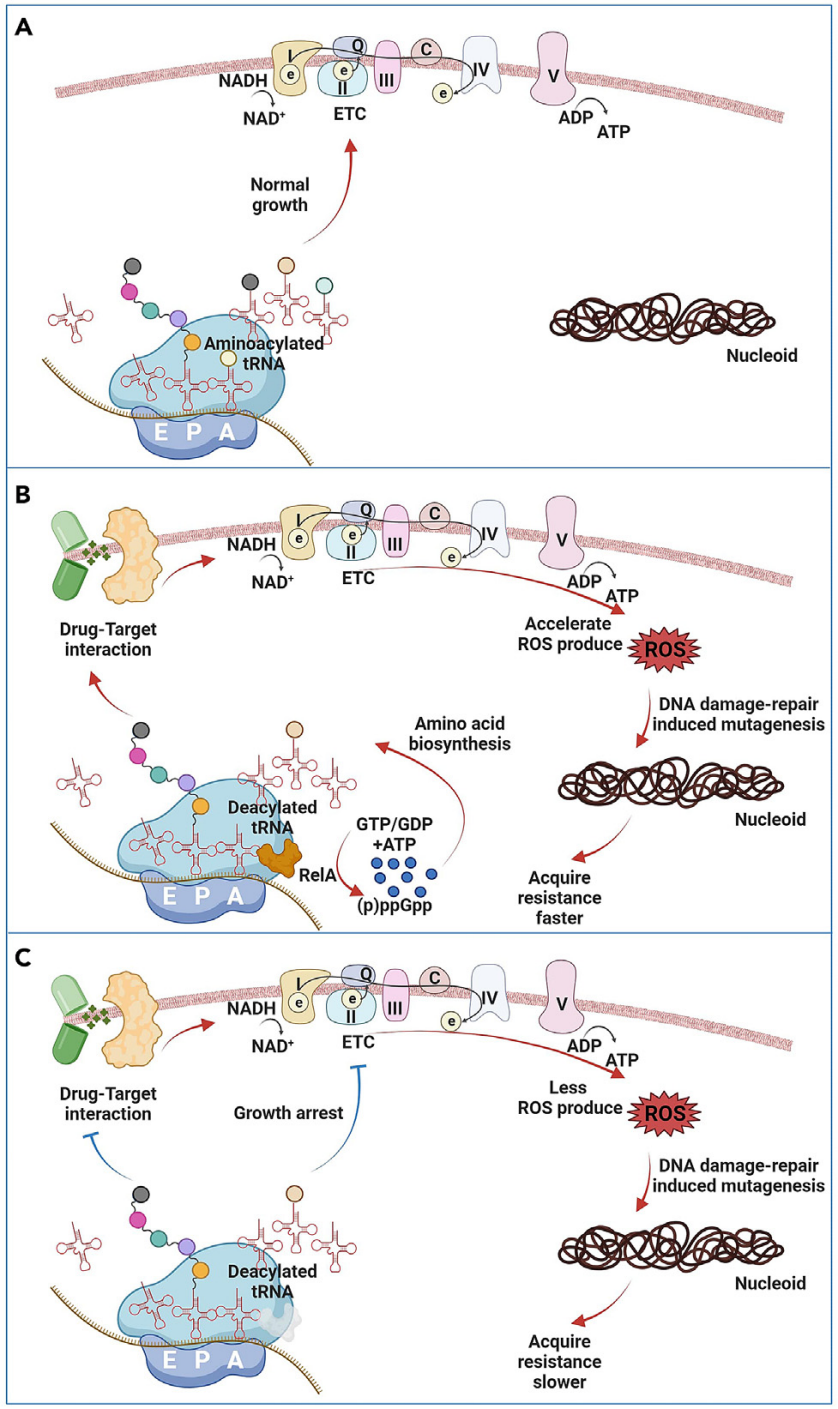
Model depicting the role of RelA synthesized (p)ppGpp on *de novo* acquisition of antibiotic resistance (A) Aminoacylated tRNA is provided to synthesize proteins during translation elongation to maintain normal growth and reproduction. (B) In response to antibiotics, cells synthesize additional proteins, which leads to insufficient aminoacylated tRNAs. Deacylated tRNAs binding to ribosome triggers (p)ppGpp to regulate amino acid synthesis, thereby reacting to antibiotics. Bactericidal antibiotics and the cell target interactions generate sub-lethal levels of ROS as a by-product through the accelerated operation of the ETC. Mutagenesis by ROS contributes to the development of drug resistance. (C) Knockout of *relA* results in sustained amino acid starvation. Cellular responses to antibiotics and production of ROS will be reduced. The development of drug resistance is slowed down.

In this experimental design, the single gene knockout of *relA* or *rpoS* decelerated the acquisition of resistance upon exposure to bactericidal antibiotics. This slowdown may be attributed to the regulatory function of (p)ppGpp and the mutagenic effect of ROS (Figure 4). Under normal conditions without antibiotic exposure, the growth rate of the naive wild-type and Δ*relA* cells was roughly the same (Figure 2D). Bacteria can intrinsically synthesize various amino acids even in a minimal medium, ensuring sufficient aminoacylated tRNAs for translation elongation and maintaining normal growth and reproductive functions (Figure 4A). Bacteria employ multiple strategies to respond to antibiotics, including the synthesis of enzymes to degrade antibiotics, reducing drug entry, increasing drug efflux, and enhancing target modification.^49^ This increased synthesis of corresponding proteins leads to higher levels of emerging deacylated tRNA. Binding of deacylated tRNAs to the A site of the ribosome leads to an interruption of the translation elongation process.^50^ However, RelA can recognize deacylated tRNAs bound to ribosomes and then synthesize (p)ppGpp, temporarily redirecting transcription from growth-related genes to genes involved in stress resistance and starvation survival.^51^^,^^52^ In addition, (p)ppGpp can also increase the amount of aminoacylated tRNAs through amino acid synthesis and proteolysis, thereby ensuring an effective bacterial antibiotic stress response.^53^ During exposure to bactericidal antibiotics, the drug-target interactions stimulate the acceleration of the electron transport chain, resulting in the formation of by-product ROS.^17^^,^^54^ ROS-induced DNA damage and cell repair will elevate the mutation rate, thereby creating a larger window of opportunity for beneficial mutations to arise, thus accelerating the formation of antimicrobial resistance during prolonged antibiotic exposure^13^^,^^15^^,^^46^ (Figure 4B). When the *relA* gene is knocked out, the absence of RelA-induced (p)ppGpp synthesis can lead to the persistence of deacylated tRNAs.^55^ Due to the hysteresis intrinsic synthesis of amino acids in minimal media, this reduces the cell’s ability to respond effectively to antibiotics, impairs its capacity to sustain growth, and weakens activity of the electron transport chain. Consequently, ROS production falls below a tipping point that is beneficial for increased non-lethal mutation rates, thus hindering drug resistance formation (Figure 4C).

As a global regulator, (p)ppGpp interacts with the DnaK repressor (DksA) by directly binding RNA polymerase and influencing the transcription of specific genes.^56^ In addition, (p)ppGpp modulates several sigma factors, such as the RNA polymerase sigma factor RpoS, which guides RNA polymerase transcription in response to specific stress conditions.^52^ RpoS serves as the master transcriptional regulator of the general stress response, being involved not only in starvation response but also in various other responses, including pH changes, oxidative stress, high temperature, and osmotic pressure.^57^ Importantly, RpoS promotes the generation of SIM, enabling cells to improve their environmental adaptability through evolution.^58^ Upon exposure to antibiotics, RpoS enhances the transcription of low-fidelity DNA polymerases in response to DNA damage.^59^ This increases the mutation rate during the repair process, potentially contributing to the formation of antimicrobial resistance.^46^^,^^47^ The resistance evolutions of the *rpoS* knockout strain were slower than that of the wild type, which aligns with the aforementioned theory (Figures 1E–1H). HipA and HipB belong to the type II TA system.^11^ HipA can phosphorylate the glutamate (Glu) tRNA ligase (GltX), which results in the accumulation of uncharged tRNA (Glu), subsequently triggering the synthesis of (p)pp(G)pp by RelA.^60^ HipB serves as a transcriptional repressor that counteracts the actions of HipA, thus inhibiting the formation of high-persister cells induced by HipA in response to antibiotics.^12^^,^^61^ The resistance acquisition rates of the wild-type and Δ*hipA* strains exhibited similar patterns (Figures 1I–1L). However, the resistance evolution rate of the Δ*hipB* strains was faster than that of the wild type upon exposure to bactericidal antibiotics (Figures 1M–1P). These observations suggest that the deletion of the *hipA* gene does not affect the acquisition of resistance, whereas the absence of *hipB* weakens the neutralization of HipA, and the subsequently induced (p)ppGpp appears to accelerate the development of antibiotic resistance. Upon phosphorylation of GltX by HipA, the accumulation of uncharged tRNAs leads to the upregulation of overall amino acid synthesis induced by (p)ppGpp, thereby enhancing the cells’ tolerance to antibiotics. This contrasts with the deceleration of resistance acquisition observed in the Δ*relA* strains, yet ultimately underscores the correlation between (p)ppGpp and resistance acquisition.

Resistant cells exhibited common target-specific mutations in response to the three bactericidal antibiotics.^13^^,^^26^^,^^44^ For instance, mutations in the *ampC* gene were observed under amoxicillin treatment, *gyrA* gene mutations occurred under enrofloxacin treatment, and *fusA* gene mutations were detected under kanamycin treatment (Tables 1, 2, 3, and 4). Additionally, non-target-specific mutations in genes related to efflux pumps and antibiotic permeability reduction were also involved in the development of antibiotic resistance. Furthermore, certain genetic mutations not directly associated with antibiotic resistance drew our attention. For example, in Δ*relA*-resistant strains, frequent mutations in the *spoT* gene strongly suggested that these strains were under stringent stress. These *spoT* gene mutations may serve as compensatory evolution to regulate the synthesis of (p)ppGpp.^62^ Moreover, *dps* mutations appeared in both ΔrelA *and* ΔrpoS resistant strains following amoxicillin or enrofloxacin exposure (Tables 1 and 3). This DNA protection during starvation protein, Dps, binds to the chromosome forming a DNA-protein crystal that protects DNA from oxidative damage.^63^^,^^64^^,^^65^ However, this in turn reduces the mutagenic responses by ROS-induced DNA damage repair. In contrast, the SIM coding gene *hfq*, SOS system coding gene *sulA*, and oxidative stress inducible gene *oxyR* found in *ΔhipA* and *ΔhipB* resistant strains predicted the emergence of damage-repair-inducing mutations.

The role of ROS as a secondary killing mechanism of bactericidal antibiotics has been elucidated in recent years.^17^^,^^19^^,^^66^ ROS-induced stress is involved in *de novo* antimicrobial resistance acquisition according to the principle of hormesis.^67^^,^^68^ Knockout of ROS-removing genes accelerated resistance development, whereas the ROS scavenger thiourea attenuated it.^13^ Similarly, in this study, we observed that the knockout of *relA* resulted in reduced ROS production in resistant strains, leading to decreased production of the signaling molecule 8-HOdG associated with ROS-induced damage (Figures 2B and 2C). As one of the possible oxidative base damages, 8-HOdG arises from guanine hydroxylation at the nucleotide pool or genomic DNA level.^69^ It induces guanine-to-thymine mutations during replication as it prefers to pair with adenine instead of cytosine.^70^^,^^71^ Bacteria engage several DNA repair genes through the SOS stress system to respond to the ROS-caused DNA damage. ROS-induced DNA oxidative damage is primarily repaired through the DNA base excision repair (BER) pathway.^72^ Formamidopyrimidine-DNA glycosylase MutM is involved in the BER of DNA damaged by oxidation, it recognizes lesions such as 8-HOdG or thymine glycol and removes them.^73^ We found that the transcription levels of *mutM* were lower in Δ*relA*-resistant strains compared to WT-resistant strains after exposure to amoxicillin or enrofloxacin (Figure 3). Additionally, the error-prone DNA polymerase coding genes *umuCD* exhibited a lower transcription level in Δ*relA*-resistant strains after exposure to amoxicillin or kanamycin. This low-fidelity polymerase V lacks intrinsic 3′–5′ exonuclease proofreading activity, and thus easily induces mutations during DNA repair.^74^ This appears to act as a protective mechanism by increasing the generation of resistance-associated mutations, which were reduced after the knockout of *relA*. Changes in the expression levels of genes related to DNA repair were found upon exposure to non-DNA-specific targeting antibiotics, such as amoxicillin and kanamycin. This observation further underscores that a shared mutagenic pathway is attributable to ROS.

### Limitations of the study

Our research connects the stringent stress response together with the oxidative stress response, suggesting, but not decisively proving that RelA-synthesized (p)ppGpp plays a crucial role in antimicrobial resistance development by regulating bacterial growth rate and ROS formation. This study shows that although high levels of ROS are lethal, moderate levels of ROS enhance the rate of resistance acquisition by increasing damage-induced mutagenesis. Therefore, the principle of hormesis applies: high levels of stress caused by exposure to high concentrations of antibiotics are lethal, but low-level exposure induces resistance mutations, making the cell resistant, which is beneficial. The intracellular mechanism is inactive when the synthesis of (p)ppGpp is limited, the production of by-product ROS is reduced, ultimately slowing down the development of antibiotic resistance. These results suggest potential strategies to reduce resistance development, such as the design of (p)ppGpp inhibitor relacin and its analogs.^75^^,^^76^ However, the specific ROS level that reduces the development of resistance needs to be further elucidated.

## STAR★Methods

### Key resources table

**Table:** 

REAGENT or RESOURCE	SOURCE	IDENTIFIER
**Bacterial and virus strains**		

*E. coli* K12 MG1655	Lab collection	N/A
*E. coli* K12 Δ*relA*	CGSC	JW2755
*E. coli* K12 Δ*rpoS*	CGSC	BW28465
*E. coli* K12 Δ*hipA*	CGSC	JW1500-2
*E. coli* K12 Δ*hipB*	CGSC	JW1501-1

**Chemicals, peptides, and recombinant proteins**		

Sodium phosphate monobasic dihydrate	Sigma-Aldrich	71500
Potassium chloride	Sigma-Aldrich	P5405
Magnesium chloride hexahydrate	Sigma-Aldrich	M2393
Ammonium chloride	Sigma-Aldrich	A9434
Sodium sulfate	Merck	1.06649
Titriplex I	Merck	1.08416
Glucose	Sigma-Aldrich	D9434
Amoxicillin	Sigma-Aldrich	A8523
Enrofloxacin	Sigma-Aldrich	17849
Kanamycin	Duchefa Biochemie	K0126
Tetracycline	Sigma-Aldrich	T3258
HPF	Invitrogen	H36004

**Critical commercial assays**		

DNeasy Blood & Tissue Kits	Qiagen	69504
NEBNext Ultra II FS DNA Library Prep Kit	New England BioLabs	E7805L
NEBNext Multiplex Oligos	New England BioLabs	E7335L
RNeasy Protect Bacteria Kit	Qiagen	74524
NEBNext rRNA Depletion Kit	New England BioLabs	E7850L
NEBNext Ultra II Directional RNA Library Prep Kit	New England BioLabs	E7760S
DNA Damage Competitive ELISA Kit	Invitrogen	EIADNAD

**Deposited data**		

Whole gene sequencing raw data	This study	BioProject PRJNA954686, PRJNA1019139, PRJNA1019238, PRJNA1019265, PRJNA1019295
RNA-seq raw data	This study	BioProject PRJNA988039, PRJNA1019310

**Recombinant DNA**		

pCP20	Datsenko & Wanner^77^	N/A

**Software and algorithms**		

ImageJ	NIH	https://imagej.nih.gov
Prism 9	GraphPad software	https://www.graphpad.com/
Bowtie2	Langmead & Salzberg^78^	http://bowtie-bio.sourceforge.net/bowtie2/index.shtml
Freebayes	Garrison & Marth^79^	https://github.com/freebayes/freebayes
Lofreq	Wilm et al.^80^	https://sourceforge.net/projects/lofreq/
Snpeff	Cingolani et al.^81^	https://pcingola.github.io/SnpEff/
IGV	Robinson et al.^82^	https://igv.org/
cn.MOPS	Klambauer et al.^83^	http://www.bioinf.jku.at/software/cnmops/
HTSeq	Anders et al.^84^	https://pypi.org/project/HTSeq/
DESeq2	Love et al.^85^	http://www.bioconductor.org/packages/release/bioc/html/DESeq2.html

**Other**		

NextSeq 550 System	Illumina	https://emea.illumina.com/systems/sequencing-platforms/nextseq.html

### Resource availability

#### Lead contact

Further information and requests for resources and reagents should be directed to and will be fulfilled by the lead contact, Benno ter Kuile (b.h.terkuile@uva.nl).

#### Materials availability

All antibiotic resistance strains generated in this study can be requested from the lead contact.

#### Data and code availability

• The binary alignment/map (BAM) files of the whole gene sequencing and RNA sequencing raw data have been archived in the NCBI database and are available for access through the BioProject PRJNA954686 (WT), PRJNA1019139 (Δ*relA*), PRJNA1019238 (Δ*rpoS*), PRJNA1019265 (Δ*hipA*), PRJNA1019295 (Δ*hipB*), and PRJNA988039 & PRJNA1019310 (RNAseq).
• This paper does not report original code.
• Any additional information required to reanalyze the data reported in this paper is available from the lead contact upon request.

### Experimental model and subject details

#### Bacterial strains, growth media, and culture conditions

The antibiotic-sensitive single-gene-knockout *E. coli* K12 strains Δ*relA*, Δ*rpoS*, Δ*hipA*, and Δ*hipB*, and the wild-type MG1655 were cultured in a phosphate buffered (100 mM NaH_2_PO_4_ · 2H_2_O) defined minimal Evans medium supplemented with 55mM glucose (pH 6.9).^86^ The kanamycin resistance cassette in knockout strains was substituted with temperature-sensitive pCP20 plasmid.^77^ The cultures were incubated in 10 mL tubes at a temperature of 37°C and constantly shaken at 200 rpm.

### Method details

#### Evolution experiments and MIC determination

*De novo* resistance acquisition evolution experiments were executed following established protocols.^87^ In brief, a single clone of each strain was cultured in tube contained Evans medium overnight, and an appropriate volume of each culture was inoculated into a fresh medium resulting in an initial optical density (OD) at 600 nm of 0.1. Antibiotics were added to the respective cultures at a concentration of one-fourth of the MIC and incubated overnight. A control group without antibiotics was also maintained. If, on a subsequent day, the OD_600_ of the antibiotic-treated culture exceeded 75% of the antibiotic-free culture, a portion of this culture was transferred to a fresh medium at an OD_600_ of 0.1. The antibiotic concentration was then doubled and maintained in two separate tubes. On the third day, if the OD_600_ of the high-concentration antibiotic-treated culture surpassed 75% of the low-concentration antibiotic culture, a portion of the high-concentration culture was transferred to a fresh medium. Otherwise, the low-concentration antibiotic culture was chosen. This process continued with an incremental doubling of the antibiotic concentration until stable resistant strains were established. Cultures without antibiotics were continuously incubated daily throughout the evolution experiment as a control. Each strain's evolution experiment was independently replicated at least twice.

MIC testing was performed three times a week using a spectrophotometer plate reader (Thermo Fisher Scientific) to monitor resistance development. Cultures with an initial OD_600_ of 0.05 were incubated in 150 μL of medium within a 96-well plate. Antibiotic concentrations ranged from 0.5 to 2048 μg/mL, with two-fold increment steps. Following overnight incubation, the MIC was defined as the lowest concentration with a final OD_600_ below 0.2. MIC values at day 10, day 20, and day 30 for each strain against antibiotics were documented during the evolution experiments. Each biological replicate contains three technical replicates. The data were presented as means ± SD, statistical significance was determined using a one-way ANOVA, ∗p < 0.05, ∗∗p < 0.01.

#### Whole genome sequencing

Genomic DNA from each final resistant strain and its corresponding antibiotic-free strains was extracted using the DNeasy Blood and Tissue Kit (Qiagen). Subsequently, whole-genome sequencing was executed utilizing the NextSeq 550 next-generation sequencing system (Illumina). Sequencing analysis followed established protocols,^13^ encompassing the alignment of reads to reference genomes via Bowtie2. Variant calling utilized Freebayes and Lofreq, while Snpeff facilitated variant annotation. Shared mutations between resistant strains and their antibiotic-free strains were excluded. Specific single nucleotide polymorphisms (SNPs) and small insertions/deletions (indels) were documented. *ampC* amplification regions were identified through copy number analysis using cn.MOPS.^83^

#### RNA sequencing

Messenger RNA from the wild-type and Δ*relA* resistant strains was extracted using the RNeasy Protect Bacteria Kit (Qiagen). The RNA sequencing and data analysis were according to the previous protocols.^13^ Similar to whole genome sequencing, reads were aligned to reference genomes using Bowtie2. To assess differential gene expression, normalized gene expression Log_2_ fold change values were calculated using HTSeq and DESeq2 by comparing resistant strains to antibiotic-free controls. Log_2_ fold change values greater than 2 in each treatment group were selected and clustered according to the Gene Ontology Biological Process (GO-BP). Clustered genes were summarized into relative abundance results based on whole genome sequencing results, and genes related to DNA repair were represented in a heat map.

#### ROS measurements

The fluorescent dye HPF (Sigma) was employed to quantify the level of ROS production.^88^ Strains treated with antibiotics were cultivated to the early-log-phase and subsequently inoculated 10 μM HPF into culture for 40 minutes. The detection of ROS-positive populations was conducted using the BD FACSAria™ III Sorter. Each high-resistant sample was independently replicated three times.

#### 8-HOdG measurements

Measurement of the 8-HOdG level was carried out using the DNA Damage Competitive ELISA Kit, following established protocols.^13^ Briefly, experimental strains were grown to the early-log-phase and subsequently pelleted in 0.3 mL of lysis buffer (containing 10 mM Tris-HCl, 2 mM EDTA, 1% SDS). A five-fold dilution of each sample was loaded onto the antibody-coated 96-well plate, followed by incubation as recommended. The absorbance at OD_450_ was measured using a spectrophotometer plate reader (Thermo Fisher Scientific). A standard curve was constructed to determine the 8-HOdG level of each sample. DNA concentration was detected using a microvolume spectrophotometer (DeNovix) and utilized for normalization. Each measurement on high-resistant samples was independently replicated three times.

#### Growth rate measurements

The wild-type and Δ*relA* final resistant strains were treated with the maximum concentrations of each antibiotic at which they could still grow and cultured overnight in a 96-well plate. Growth curves were generated using a spectrophotometer plate reader and absorbance was recorded at 10-minute intervals. Growth rates were calculated by the Growthrates-in-R (https://github.com/Pimutje/Growthrates-in-R/releases/tag/Growthrates). Measurements on each high-resistant sample were independently replicated three times.

### Quantification and statistical analysis

Statistical analysis was performed using IBM SPSS statistical software. Details on the statistical methods employed for each experiment can be found in the figure legends and the corresponding methods.

## Supplemental information

Table S1. Gene mutations in antibiotic resistant strains, related to Table 1–5
